# Conversion of cell-survival activity of Akt into apoptotic death of cancer cells by two mutations on the BIM BH3 domain

**DOI:** 10.1038/cddis.2015.118

**Published:** 2015-07-02

**Authors:** J-S Kim, B Ku, T-G Woo, A-Y Oh, Y-S Jung, Y-M Soh, J-H Yeom, K Lee, B-J Park, B-H Oh, N-C Ha

**Affiliations:** 1 Department of Agricultural Biotechnology, Center for Food Safety and Toxicology, Research Institute for Agriculture and Life Sciences, Seoul National University, Seoul, Republic of Korea; 2 Department of Biological Sciences, KAIST Institute for the Biocentury, Cancer Metastasis Control Center, Korea Advanced Institute of Science and Technology, Daejeon, Republic of Korea; 3 Functional Genomics Research Center, Korea Research Institute of Bioscience and Biotechnology, Daejeon, Republic of Korea; 4 Department of Molecular Biology, Pusan National University, Busan, Republic of Korea; 5 Department of Life Science, Chung-Ang University, Seoul, Republic of Korea

## Abstract

Survival and proliferation of cancer cells are often associated with hyperactivity of the serine/threonine kinase, Akt. Herein, we show that prosurvival activity of Akt can be converted into prodeath activity by embedding an Akt recognition sequence in the apoptogenic BH3 domain of human BIM. The recognition sequence was created by introducing two mutations, I155R and E158S, into the core region of the BIM BH3 domain. Although a 21-mer BIM BH3 peptide containing these two mutations bound weakly to BCL-X_L_ and BCL-2, this peptide with phosphorylation of Ser158 bound to these proteins with a dissociation constant of <10 nM. The crystal structure of the phosphorylated peptide bound to BCL-X_L_ revealed that the phospho-Ser158 makes favorable interactions with two BCL-X_L_ residues, which cannot be formed with unphosphorylated Ser158. Remarkably, the designed peptide showed a cytotoxic effect on *PTEN*-null PC3 tumor cells whose Akt activity is aberrantly high. The cell-killing activity disappeared when the cellular Akt activity was lowered by ectopic PTEN expression. Thus, these results lay a foundation for developing a peptide or protein agent that is dormant in normal cells but is transformed into a potent apoptogenic molecule upon phosphorylation by hyperactivity of Akt in cancer cells.

The interplay between the BCL-2 family proteins regulates mitochondrion-mediated apoptotic cell death.^1, 2^ The BCL-2 family proteins are characterized by having at least one BCL-2 homology (BH) domain, and they are classified into three distinct subgroups based on their functional and structural features. One subgroup consists of BAX and BAK, which contain the BH1-BH4 domains and mediate apoptosis by increasing the permeability of the mitochondrial outer membrane (MOM) and thus leading to the release of the apoptogenic factors, such as cytochrome *c* and Smac/Diablo.^3, 4, 5, 6^ Another subgroup is composed of antiapoptotic proteins, BCL-2, BCL-X_L_, BCl-w, MCL-1, A1 and BCL-B, which contain the BH1-BH4 domains that are arranged to form an extended hydrophobic groove known as the BH3-binding groove.^7^ The remaining subgroup is composed of a diverse set of proteins that are unrelated to each other except for the possession of the BH3 domain.^7^ These BH3-only proteins sense and convey apoptotic cell death signals, ultimately leading to the activation of BAX and BAK.^8, 9^ The antiapoptotic BCL-2 subfamily proteins bind the BH3 domain of BAX/BAK and of the BH3-only proteins through their BH3-binding groove.^10, 11, 12, 13, 14, 15^

Biochemical studies have discovered that a number of the BH3-only proteins termed ‘activators', such as BID and BIM, bind directly to BAX and induce its activation, whereas other BH3-only proteins termed ‘sensitizers' induce apoptosis by releasing the activators sequestered by the antiapoptotic proteins.^5, 16, 17^ A recent crystallographic study revealed that the BID BH3 peptide binds to the canonical BH3-binding groove of BAX and induces a pronounced conformational change that exposes the BH3 domain of BAX.^18^ The activated BAX oligomerizes to induce the permeabilization of the MOM.^6^ The antiapoptotic BCL-2 proteins were suggested to sequester the BH3 domains of both BAX and the activator BH3-only proteins to prevent the BAX oligomerization.^18^

Apoptosis is attenuated in cancer cells because of the abundance of antiapoptotic BCL-2 proteins and/or prevention of apoptosis induction. Anticancer BH3 peptides have been developed, especially those derived from BIM, which interacts with all of the antiapoptotic proteins with extremely high affinity.^15, 19^ These BH3 peptides exhibit a broad and multimodal targeting of the BCL-2 family proteins.^20, 21, 22^ Promising small molecular anticancer compounds have also been developed that mimic the BH3 peptides and bind to the surface groove of the antiapoptotic proteins.^23^ ABT-737 and ABT-263 selectively bind to and lower the amounts of the functional BCL-2, BCL-X_L_ and BCL-w proteins to induce the apoptotic death of tumor cells that depend especially on the overexpression of the three proteins.^24, 25^ The BH3 peptides and the BH3 mimetics both bear an intrinsic shortcoming in that they inhibit the BCL-2 family proteins not only in cancer cells but also in normal cells as they cannot distinguish cancerous from normal cells.

One of the hallmarks of many cancer and tumor cells is the hyperactivation of the serine/threonine (Ser/Thr) protein kinase Akt, which is a key signaling molecule in the cellular survival pathway.^26^ In many types of cancers, including glioma, prostate cancer and breast cancer, Akt is required to maintain a proliferative state and for progression into a more malignant state in conjunction with genetic mutations.^26, 27, 28^

We set out to develop a molecule that can respond to the hyperactivity of Akt and can lead to the death of cancer cells. Herein, we describe the embedment of the Akt recognition sequence into the BIM BH3 peptide and the cancer cell-specific apoptogenic property of the resulting BIM BH3 peptide variant characterized by X-ray crystallography, calorimetry and cell-based biochemistry.

## Results

### Design of a BIM BH3 peptide with an Akt recognition sequence

**Figure 1:**
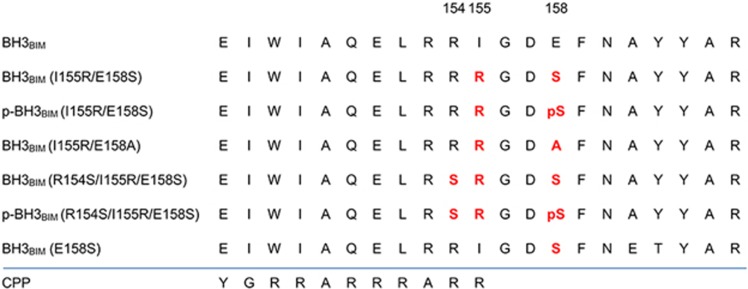
Amino acid sequences of the peptides used in this study. The substituted residues are in red, and ‘pS' stands for the phosphorylated serine residue

We chose the BIM BH3 as the template sequence for mutagenesis. According to the crystal structure of the mouse BIM BH3 domain bound to BCL-X_L_, 21 residues of BIM form the core region of the BH3 domain that spans the surface groove of BCL-X_L_.^14^ The 21 residues correspond to 145-EIWIAQELRRIGDEFNAYYAR-165 of human BIM, which is referred to as BH3_BIM_ (Figure 1). To design a BH3_BIM_ peptide variant that can be phosphorylated by Akt, we noted Glu158 in BH3_BIM_. Glu158 is not a strictly conserved residue, but is involved in a polar interaction with Tyr101, a conserved residue of BCL-X_L_ in the crystal structure,^14^ suggesting that this residue contributes to the binding affinity of BH3_BIM_. Thus, it was expected that a BH3_BIM_ variant containing serine in place of Glu158 would exhibit reduced binding affinity for the antiapoptotic BCL-2 family proteins, but phosphorylation of Ser158 could restore the binding affinity since phosphorylated serine often serves as a mimic of glutamate residue. Importantly, this variant peptide turns into an Akt recognition sequence by a second mutation of Ile155Arg, because the doubly mutated sequence has a RR*R*^155^GD*S*^158^F stretch that conforms to the Akt recognition sequence (RxRxx*T/S*F; *T/S*, phosphorylation site).^29^ Ile155 of human BIM, which is a strictly conserved residue, is involved in extensive hydrophobic interactions with BCL-X_L_ in the complex between mouse BCL-X_L_ and a BIM BH3 peptide.^14^ Nonetheless, a modeling experiment with this BH3_BIM_ peptide variant, referred to as BH3_BIM_(I155R/E158S), indicated that arginine in place of Ile155 is sterically compatible and does not completely abrogate the binding interaction. In addition, its guanidinium group seemed to form an intramolecular salt bridge with Glu151 in this peptide.

In particular, phosphorylated Ser158, not Ser158, of BIM appeared to form a polar interaction with Tyr101 of BCL-X_L_. Furthermore, the phosphate group on Ser158 was within the intramolecular ionic-bonding distance from the guanidinium group of Arg154. Based on these expectations, we hypothesized that unphosphorylated BH3_BIM_(I155R/E158S) would suffer from reduction in the binding affinity compared with the wild-type version, but phosphorylation of the peptide at Ser158 could provide some compensation by directly interacting with BCL-X_L_. Thus, it seemed plausible that a full-length BIM or a BH3 region bearing the I155R and E158S substitutions could be phosphorylated in cancer cells with elevated Akt activity and then be converted into a potent inhibitor against antiapoptotic BCL-2 proteins, while they are not potentiated in normal cells due to low or controlled Akt activity.

### BH3_BIM_(I155R/E158S) is phosphorylated by Akt and potently binds to antiapoptotic BCL-2 proteins

**Figure 2:**
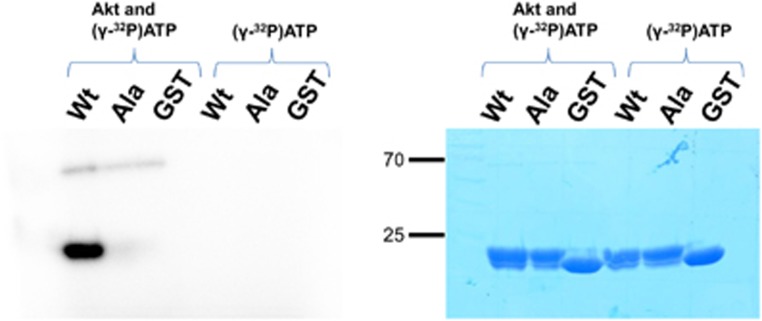
*In vitro* Akt kinase assay. GST-fused BH3_BIM_(I155R/E158S) (WT), BH3_BIM_(I155R/E158A) (Ala) or GST was incubated with ATP and [*γ*-^32^P]ATP in the presence or absence of Akt. The mixtures were resolved on a SDS-polyacrylamide gel, and the radioactivity (left panel) and Coomassie-staining (right panel) are shown. Only GST-fused BH3_BIM_(I155R/E158S) was phosphorylated

To examine whether the designed sequence is phosphorylated by Akt as we intended, we carried out an *in vitro* Akt activity assay by using GST-tagged BH3_BIM_(I155R/E158S) as the substrate in the presence of [*γ*-^32^P]ATP. GST-tagged BH3_BIM_(I155R/E158S) was efficiently phosphorylated, while GST and GST-tagged BH3_BIM_(I155R/E158A) employed as controls were not phosphorylated (Figure 2), demonstrating that Ser158 in BH3_BIM_(I155R/E158S) is specifically phosphorylated by Akt.

**Figure 3:**
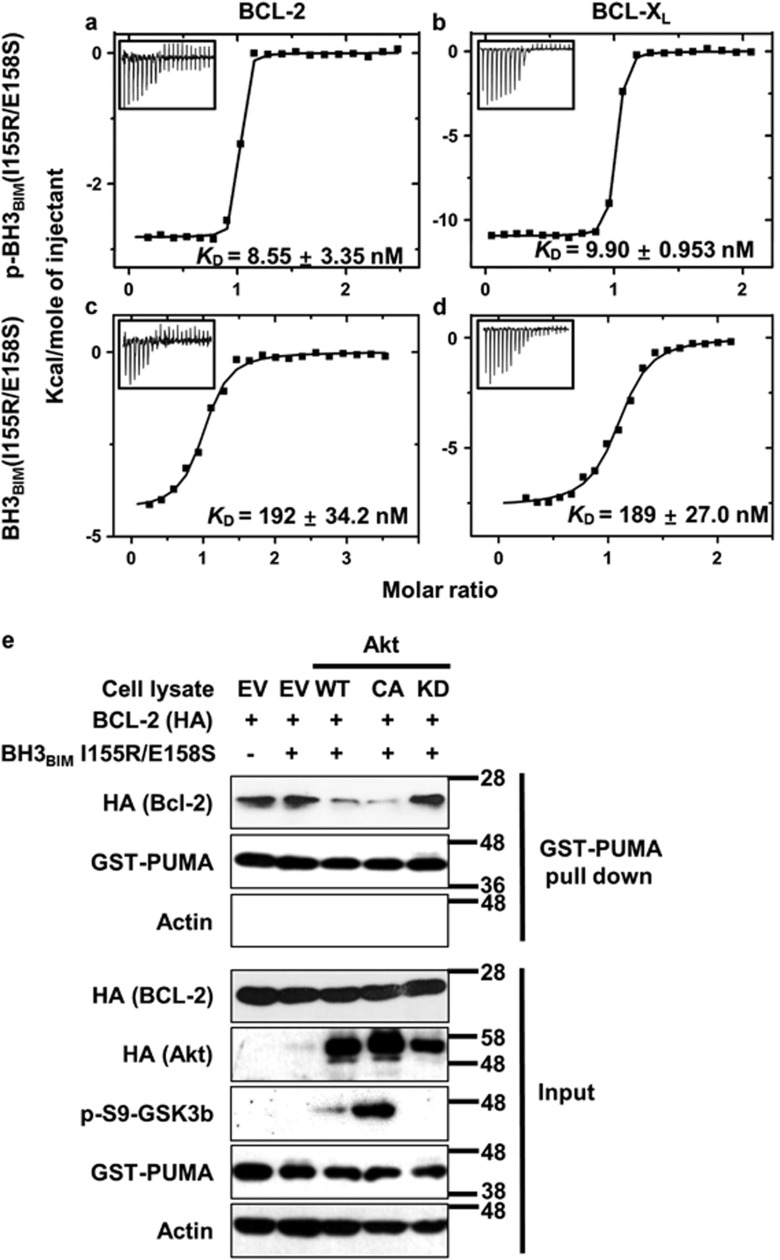
Phosphorylation-dependent binding of BH3_BIM_(I155R/E158S) to BCL-2 and BCL-X_L_. (**a–d**) The ITC analyses were carried out by titrating the indicated peptides (0.2 mM) into BCL-2 or BCL-X_L_ (20 *μ*M). The *K*_D_ values were deduced from curve fittings of the integrated heat per mole of added ligand (insets). (**e**) Competition assay. The BH3_BIM_(I155R/E158S) peptide was incubated with cell lysate containing overexpressed Akt (wild type (WT), constitutively active form (CA) or kinase-dead (KD) mutant) and HA-tagged BCL-2 protein. This mixture was incubated with GST-PUMA bound to glutathione agarose resin. After washing, bound HA-tagged BCL-2 was detected by immunoblotting. Detection of p-S9-GSK3*β* was to monitor the Akt activity. Input: used cell lysates and GST-PUMA. EV: empty vector transfection. Numbers: approximate molecular weight

To test if phosphorylated BH3_BIM_(I155R/E158S) binds to the BCL-2 family proteins more tightly than its unphosphorylated version, we produced recombinant BCL-2 and BCL-X_L_ proteins, and also prepared two 21-mer synthetic peptides: BH3_BIM_(I155R/E158S) and phosphorylated BH3_BIM_(I155R/E158S) at Ser158, which is referred to as p-BH3_BIM_(I155R/E158S) (Figure 1). Quantification of the binding affinities by isothermal titration calorimetry (ITC) showed that p-BH3_BIM_(I155R/E158S) interacted potently with BCL-2 and BCL-X_L_ with *K*_D_ values of 8.55 and 9.90 nM, respectively (Figures 3a and b), similar to that of a longer 36-mer BIM BH3 peptide (*K*_D_ of ~7 nM).^15^ In contrast, the unphosphorylated BH3_BIM_(I155R/E158S) peptide exhibited much lower affinities for the two proteins (*K*_D_ of 192 and 189 nM, respectively) (Figures 3c and d). Thus, phosphorylated Ser158 appeared to replace the role of Glu158 in the BH3 sequence. Furthermore, the substitution of the conserved hydrophobic Ile155 seemed to be tolerated in the binding reaction, which is intriguing given the observation that an alanine substitution of the corresponding Ile81 residue in a BAK BH3 peptide resulted in a significant reduction of the binding affinity for BCL-X_L_ (*K*_D_ value changed from 0.34 to 17 *μ*M).^30^

The measured binding affinities of p-BH3_BIM_(I155R/E158S) for BCL-2 or BCL-X_L_ are comparable to or higher than those of 36-mer BH3 peptides derived from BAX and BAK (*K*_D_ of 8.1–255 nM),^15^ suggesting that the phosphorylated BH3_BIM_(I155R/E158S) sequence, but not the unphosphorylated sequence, could displace these two apoptosis mediators from the antiapoptotic BCL-2 proteins and potentiate cell death. We indirectly tested this possibility by employing PUMA and BCL-2, whose intermolecular interaction is tighter than that between BAX (or BAK) and BCL-2,^15^ to obviate complications in using full-length BAX or BAK. Recombinant human PUMA fused to GST (GST-PUMA) was produced, and HEK293 cells were prepared to transiently overexpress BCL-2 and one of the three forms of Akt: wild-type, constitutively active or kinase-dead form. Each cell lysate was incubated with BH3_BIM_(I155R/E158S) and GST-PUMA. This peptide added to the cell lysate containing the wild-type and constitutively active form of Akt abolished the binding between BCL-2 and GST-PUMA, whereas the same peptide added to the cell lysate containing the kinase-dead form of Akt did not interfere with the binding interaction (Figure 3e). The kinase activity of the Akt proteins were confirmed by examining the phosphorylation of GSK3*β*, a cellular substrate of Akt (Figure 3e). Collectively, these results indicated that Akt-phosphorylated BH3_BIM_(I155R/E158S), and the phosphorylated peptide could compete with PUMA for binding to BCL-2, whereas the unphosphorylated peptide could not.

### Structure of BCL-X_L_ in a complex with p-BH3_BIM_(I155R/E158S)

**Figure 4:**
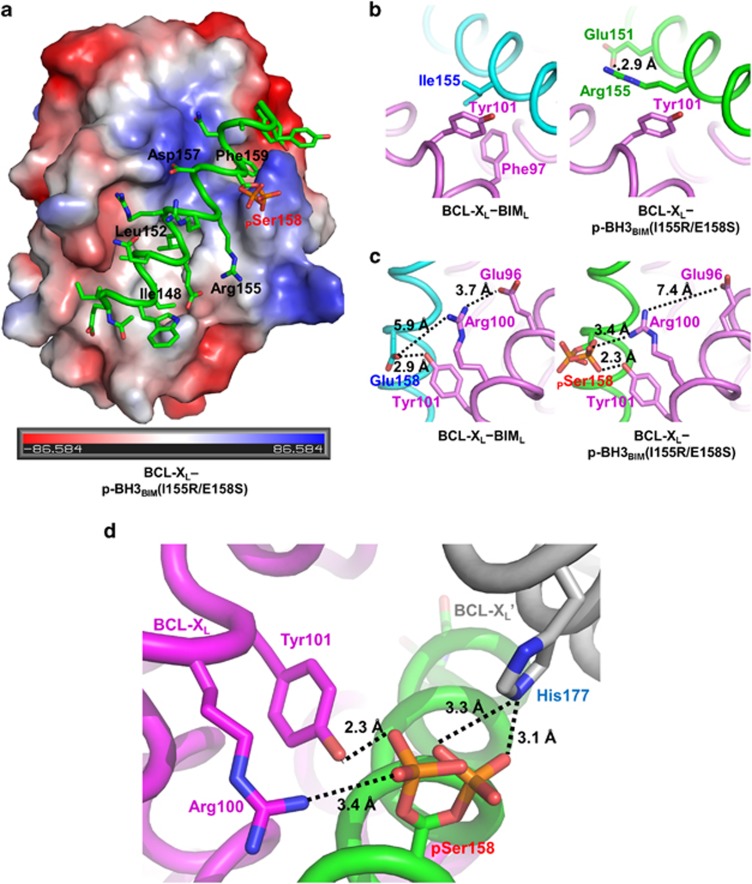
Structural analysis of the interaction between BCL-X_L_ and p-BH3_BIM_(I155R/E158S). (**a**) Crystal structure of the BCL-X_L_−p-BH3_BIM_(I155R/E158S) complex. BCL-X_L_ is shown as an electrostatic surface model with the bound peptide in green. For clarity, only the residues corresponding to the five consensus BH3 residues (black) and the phosphoserine (red) are labeled. The values for the electrostatic potential calculated using PyMOL^50^ are displayed at the bottom. (**b** and **c**) Side-by-side comparison of detailed intermolecular interactions of BCL-X_L_ with BIM_L_ (left; PDB code 1PQ1) and with p-BH3_BIM_(I155R/E158S) (right). BCL-X_L_ is in violet, and the BH3 helices are in cyan or green. (**b**) Ile155 contributing to the hydrophobic interactions (left) is substituted by arginine (right). (**c**) Phosphorylated Ser158 on the peptide is involved in electrostatic interactions with BCL-X_L_ (right). For comparison, the numbering of BIM residues is according to that of human BIM_L_. (**d**) Dual conformations of phosphorylated Ser158. The phosphoryl group of p-BH3_BIM_(I155R/E158S) interacts with Arg100 and Tyr101 of BCL-X_L_ within the complex or with His177 of the neighboring BCL-X_L_ (BCL-X_L_') in the crystal

To understand the structural basis for the critical role of the Ser158 phosphorylation, we next determined the crystal structure of BCL-X_L_ bound to p-BH3_BIM_(I155R/E158S) at a 2.1-Å resolution (Table 1). The peptide binds to the BH3-binding groove of BCL-X_L_ by forming an amphipathic *α*-helix, as observed in all of the reported structures of the BH3 peptides bound to the antiapoptotic BCL-2 family proteins^14, 15, 31^ (Figure 4a). Since the sequence of the p-BH3_BIM_(I155R/E158S) peptide is highly similar to that of the BH3 domain of mouse BIM, the presented structure can be directly compared with the structure of BCL-X_L_ bound to the BIM_L_ BH3 domain.^14^ The p-BH3_BIM_(I155R/E158S) peptide contains four of the five consensus residues that are highly conserved in the BH3 domains of the proapoptotic proteins and known to have critical roles in interacting with the antiapoptotic BCL-2 proteins. The four residues (Ile148, Leu152, Asp157 and Phe159) in the peptide are involved in the intermolecular hydrophobic or hydrophilic interactions with BCL-X_L_, similar to the corresponding residues of the BIM_L_ BH3 bound to BCL-X_L_ (not shown).

The remaining consensus residue, which corresponds to Ile155 of human BIM (Ile97 of mouse BIM), is replaced by arginine in p-BH3_BIM_(I155R/E158S). While Ile97 of mouse BIM is involved in hydrophobic interactions with Phe97 and Tyr101 of BCL-X_L_, Arg155 of p-BH3_BIM_(I155R/E158S) forms a hydrophobic interaction with Tyr101 through its hydrocarbon portion, and makes an intramolecular salt bridge with Glu151 of the peptide through its guanidinium group (Figure 4b). The salt bridge is likely to increase the helical propensity of p-BH3_BIM_(I155R/E158S) in solution and to contribute to the binding affinity of this phosphorylated peptide.

Notably, the phosphate group attached to Ser158 has a dual conformation (Figures 4c *right* and d). In one conformation, it interacts with the guanidinium group of Arg100 and the hydroxyl group of Tyr101 of BCL-X_L_, where Arg100 is distantly positioned from the carboxyl group of Glu158 (5.9Å) in the structure of the BCL-X_L_–BIM_L_ BH3 complex^14^ (Figure 4c *left*). In the other conformation, however, the phosphate group is removed from the guanidinium group (4.6Å), and instead interacts with His177 of a symmetry-related BCL-X_L_ molecule (3.1Å) (Figure 4d). Therefore, the observed conformation of phosphorylated Ser158 in the crystalline state would be different from the conformation of this residue in solution.

### Structure of BCL-X_L_ in complex with the p-BH3_BIM_(R154S/I155R/E158S) peptide

**Figure 5:**
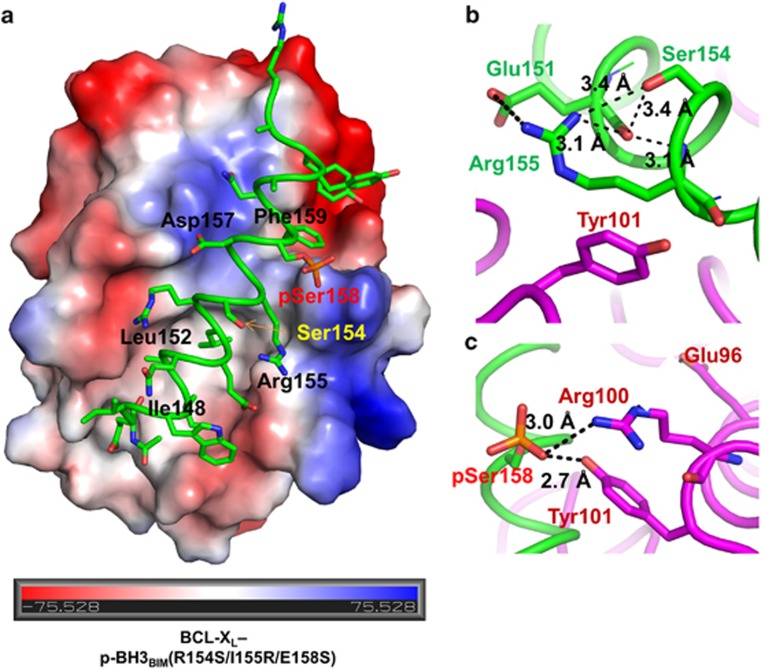
Structural analysis of the interaction between BCL-X_L_ and p-BH3_BIM_(R154S/I155R/E158S). (**a**) Crystal structure of the BCL-X_L_−p-BH3_BIM_(R154S/I155R/E158S) complex. BCL-X_L_ is shown as an electrostatic surface model with the bound peptide in green. The electrostatic potential is shown at the bottom. (**b**) Detailed interactions of the Arg155 of p-BH3_BIM_(R154S/I155R/E158S) in the complex. Polar interactions are indicated by dotted lines with the distances noted. The aliphatic chain of Arg155 has a hydrophobic interaction with the Tyr101 of BCL-X_L_. (**c**) Detailed phosphoserine-mediated intermolecular interactions

To remove the crystal packing interaction of phosphorylated Ser158, we prepared another peptide, referred to as p-BH3_BIM_(R154S/I155R/E158S), which contains an additional substitution of R154S in addition to the I155R and E158S substitutions (Figure 1). Arg154 was chosen for the substitution, as this residue in p-BH3_BIM_(I155R/E158S) was engaged in a crystal packing interaction with a symmetry mate (Supplementary Figure S1). In an ITC analysis, p-BH3_BIM_(R154S/I155R/E158S) exhibited slightly lower affinity for BCL-X_L_ (K_D_=~40 nM) compared with p-BH3_BIM_(I155R/E158S) (Supplementary Figure S2). Subsequently, we obtained crystals of BCL-X_L_ bound to this peptide in a different crystal form, and determined its structure at a 1.7Å resolution (Figure 5a and Table 1). In this crystal form, the asymmetric unit contained two copies of the complexes that exhibit nearly identical conformations (rmsd=0.063Å between 143 C*α* atoms; Supplementary Figure S3). Importantly, phosphorylated Ser158 in this peptide was not involved in the crystal packing interactions. The intermolecular interactions between BCL-X_L_ and the peptide are mostly similar to those observed in the BCL-X_L_–p-BH3_BIM_(I155R/E158S) structure (Figure 5b). In particular, phosphorylated Ser158 is in a single conformation and makes intermolecular interactions with Tyr101 and Arg100 of BCL-X_L_, which is likely to recapitulate the interactions in solution (Figure 5c). Together, the presented structures explain the significant enhancement of the binding affinity upon phosphorylation of Ser158 in the designed BH3_BIM_(I155R/E158S) peptide.

### Akt-dependent cytotoxic activity of the designed BH3_BIM_ peptide

**Figure 6:**
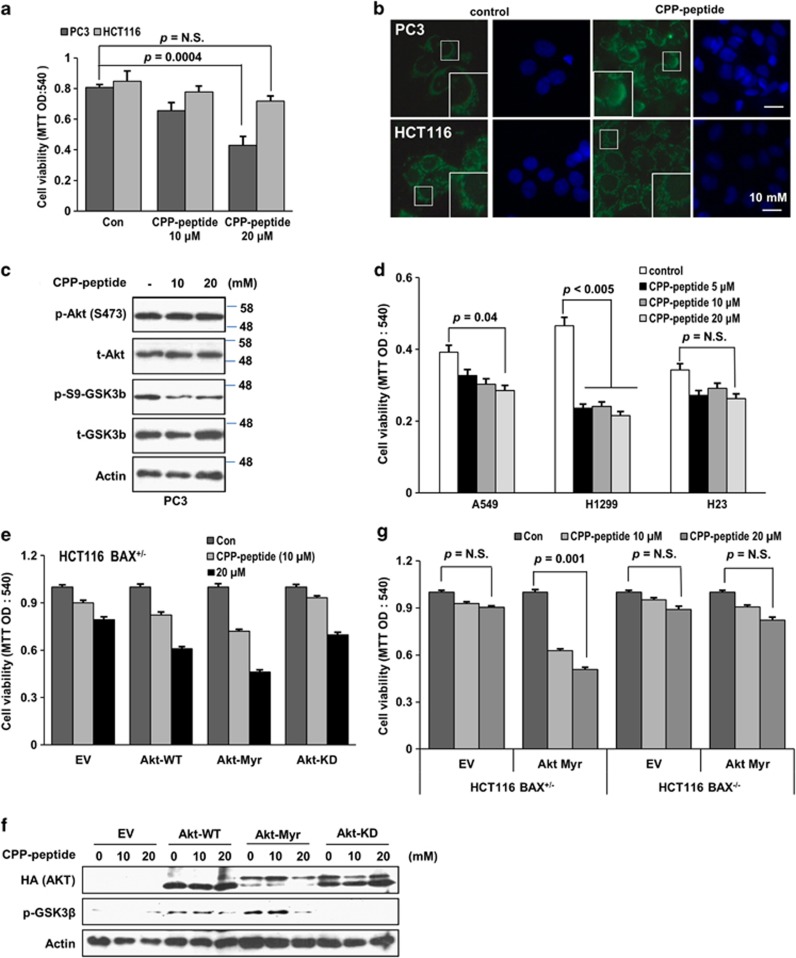
CPP-fused BH3_BIM_(I155R/E158S) peptide induces death of PC3 cells. (**a**) Cell viability evaluated by the MTT assay 72 h after treatment of the peptide (0, 10 or 20 *μ*M). N.S indicates non-significance. Error bars indicate S.D. (*n*=3). (**b**) The immunofluorescence images of PC3 and HCT116 cells treated with 20-*μ*M CPP-BH3_BIM_(I155R/E158S). Cytochrome *c* (green) was detected by anti-cytochrome *c* antibody and FITC-conjugated secondary antibody, and the nucleus was stained by DAPI. The large boxes are the enlargement of the small boxes, highlighting the cytochrome *c* staining. (**c**) Western blot (WB) analysis to examine the effect of CPP-BH3_BIM_(I155R/E158S) on the phosphorylation of Akt (at Ser473) and GSK3*β* (at Ser9). PC3 cells were incubated with the peptide for 6 h at the indicated concentration. t-Akt and t-GSK3*β* stand for total Akt and GSK3*β*, respectively. (**d**) MTT assay to examine the cytotoxic effect of CPP-BH3_BIM_(I155R/E158S) on lung cancer cell lines. The viability of *PTEN*-silenced H1299 cells was clearly reduced by the peptide. (**e**) Promotion of CPP-BH3_BIM_(I155R/E158S)-induced death of HCT116 cells by Akt. Cells were transfected with the indicated vectors for 24 h and incubated with the peptide for 72 h. Cell death was promoted by Akt-WT and Akt-Myr, but not by Akt-KD. (**f**) Phosphorylation of GSK3*β* (p-GSK3*β*) by Akt-WT and Akt-Myr, but not by Akt-KD. Cells were harvested for WB after incubation with CPP-peptide for 6 h. Actin was used for loading control. (**g**) CPP-BH3_BIM_(I155R/E158S) induced apoptosis in a BAX-dependent manner. HCT116 cells and its isogenic *BAX*^−/−^ cells were transfected with *Akt-Myr* for 24 h and incubated with the peptide at the indicated concentration for 72 h. HCT116 *BAX*^+/−^ cells, but not HCT116 *BAX*^−/−^ cells, were affected by the peptide

Next, we tested whether the BH3_BIM_(I155R/E158S) peptide exhibits cytotoxic activity. For intracellular delivery, BH3_BIM_(I155R/E158S) was fused to the C-terminus of the cell penetration peptide (CPP) derived from HIV Tat (Figure 1). PC3 and HCT116 cells were treated with the CPP-BH3_BIM_(I155R/E158S) peptide, and the MTT assay was performed. PC3 cells, which are derived from prostate cancer cells, exhibit a significantly elevated Akt activity due to the loss of PTEN, a negative regulator of Akt,^32, 33^ whereas the colon cancer-derived HCT116 cells exhibit a normal level of Akt activity. Seventy-two hours after the treatment with the fusion peptide, a strong cytotoxic activity was observed in the PC3 cells, but not in the HCT116 cells (Figure 6a). Immunofluorescence analysis suggested that the PC3 cells underwent apoptotic cell death (Figure 6b). Cytochrome *c* diffused in the cytoplasm and gradually accumulated in the nucleus, which is known to occur during apoptosis.^34^ In contrast, HCT116 retained the puncta staining pattern under the same treatment (Figure 6b). We then measured the activity of Akt by examining the phosphorylation state of itself and its substrate GSK3*β*. The CPP-BH3_BIM_(I155R/E158S) peptide reduced the phosphorylation of GSK3*β*, but not Akt itself (Figure 6c), indicating that this peptide could have acted as a substrate of Akt. To elaborate this observation, we examined the effect of the fusion peptide in three different human lung cancer cell lines. The *PTEN*-silenced H1299 cell lines exhibited sensitivity to the peptide, and the A549 cell line, which possesses a K-Ras mutation, showed a moderate response (Figure 6d). In contrast, the H23 cell line, which is derived from lung cancer cells with wild-type K-Ras and normal level of Akt activity, did not respond to this peptide, suggesting that the CPP-BH3_BIM_(I155R/E158S)-induced cell death might depend on the Akt activity. We then assessed the effect of the Akt activity by ectopically expressing wild-type Akt (Akt-WT), constitutively active Akt (myristoylated Akt; Akt-Myr) or kinase-dead-Akt (Akt-KD). Both Akt-WT and Akt-Myr promoted death of HCT116 cells, whereas Akt-KD did not show an additive effect (Figure 6e). The kinase activities of the three Akt versions were confirmed by examining GSK3*β* phosphorylation (Figures 6f and p-GSK3*β*). To know whether CPP-BH3_BIM_(I155R/E158S)-induced cell death might depend on BAX, the HCT116 *BAX*^+/−^ and the HCT116 *BAX*^−/−^ cell lines were transfected with an expression vector encoding *AKT-Myr* and the cells were treated with the peptide. Notably, the *BAX*^+/−^ cells, but not the *BAX*^−/−^ cells, were sensitized to the peptide (Figure 6g), indicating that the CPP-BH3_BIM_(I155R/E158S) peptide induced intrinsic apoptotic cell death via BAX. The survival of the *BAX*^−/−^ cells despite the CPP-BH3_BIM_(I155R/E158S) treatment could be explained by an observation that the designed peptide only weakly binds to MCL-1 (not shown), which is a major inhibitor of BAK.^35^ Presumably, both MCL-1 and BAK are expressed in these cells.^36^

### PTEN-dependent cytotoxic effect of the designed BH3_BIM_

**Figure 7:**
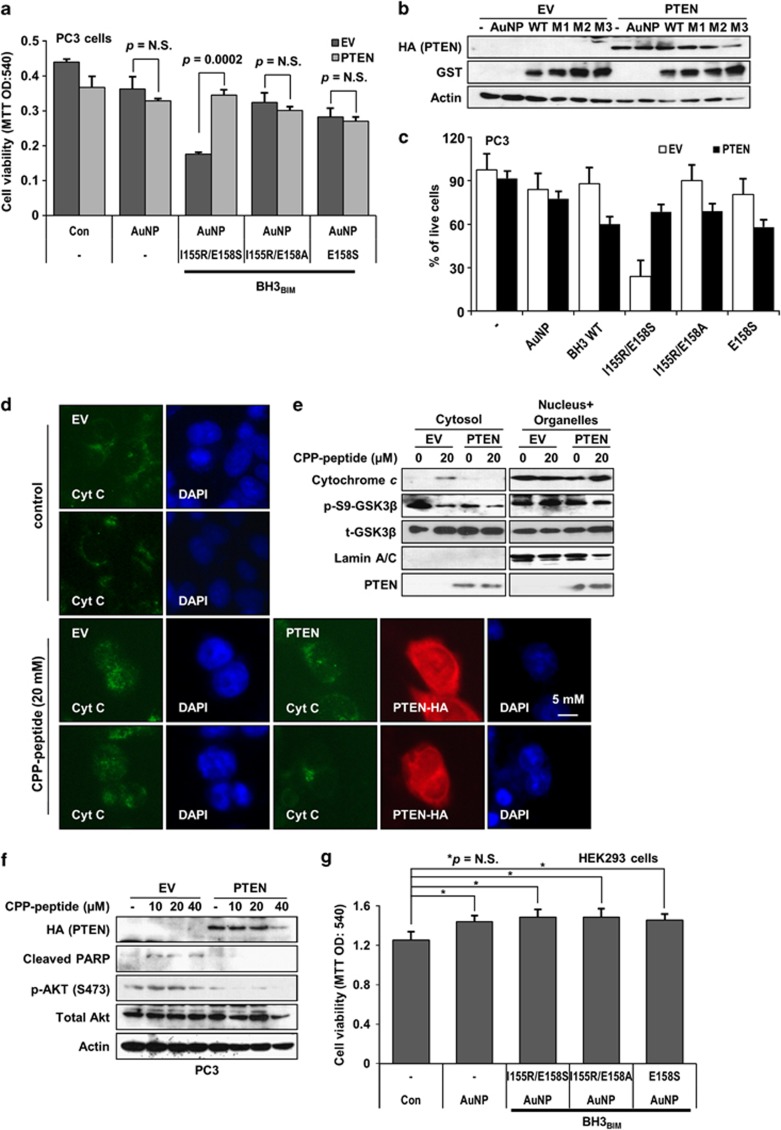
The BH3_BIM_(I155R/E158S) sequence induces phosphorylation-dependent cell death. (**a**) Cell viability by the MTT assay. One day after transfection of PC3 cells with an empty vector (EV) or a PTEN-expressing vector (PTEN), cells were treated for 12 h with AuNP-anti-GST DNA aptamer composite coated with the indicated BH3_BIM_ peptide fused to GST (5 *μ*M). (**b**) Intracellular delivery of the GST-fused peptides. Cells were washed with PBS three times to remove proteins attached to the plasma membrane. M1 stands for BH3_BIM_ (E158S), M2 for BH3_BIM_ (I155R/E158A), and M3 for BH3_BIM_ (I155R/E158S). (**c**) Cell viability by trypan blue dye exclusion assay. To confirm the effect in (**a**), cells were incubated with indicated materials for 24 h and stained with trypan blue for 10 min. Percent of live cells were determined by average of 200 cells in three independent experiments. (**d**) Immunostaining of cytochrome *c* in PC3 cells. Cells were transfected (EV or PTEN), and incubated with the CPP-BH3_BIM_(I155R/E158S) peptide (CPP-peptide) for 48 h. Cytoplasmic puncta staining pattern was observed with PC3 cell expressing PTEN (red). In contrast, a diffused nuclear pattern was observed with EV-transfected cells. (**e**) PTEN blocked the cytochrome *c* release from mitochondria. Cells were incubated with CPP-BH3_BIM_(I155R/E158S) for 6 h and subjected to cell fractionation. Increase of cytoplasmic cytochrome *c* by CPP-BH3_BIM_(I155R/E158S) was obviously blocked by PTEN expression. Reduction of p-S9-GSK3*β* indicated competition of S9-GSK3*β* with the peptide for phosphorylation by PTEN. (**f**) PTEN also blocked PARP cleavage, an indicator of caspase activation. (**g**) HEK293 cells were treated in the same way as in (**a**). Viable cells were evaluated 48 h after the treatment by the MTT assay. Error bars indicate the S.D. (*n*=3)

For efficient cytoplasmic delivery of the proteins, we applied a novel technique of using gold nanoparticles (AuNPs) coated with anti-GST DNA aptamers.^37^ To apply this technique, GST-fused the BH3_BIM_ peptides were produced and incubated with AuNPs coated with anti-GST DNA aptamer. We observed that GST-BH3_BIM_(I155R/E158S) efficiently killed PC3 cells (Figure 7a). In contrast, the other variants, GST-BH3_BIM_(E158S) and GST-BH3_BIM_(I155R/E158A), did not exhibit a cell-killing effect (Figure 7a), presumably because these two peptides lack either the Akt recognition sequence or a site for phosphorylation by Akt. Intriguingly, when the *PTEN* gene was introduced into PC3 cells by transfection, the cell-killing effect of GST-BH3_BIM_(I155R/E158S) was significantly reduced (Figure 7a). We confirmed that GST-BH3_BIM_-coated AuNPs were effectively incorporated into the cells (Figure 7b). To confirm the cytotoxic effect of each protein, we performed the trypan blue dye exclusion assay and counted viable cells. Consistent with the MTT assay described above, the AuNPs coated with GST-BH3_BIM_(I155R/E158S) clearly reduced the viability of PC3 cells (Figure 7c). In a control experiment, BH3_BIM_(I155R/E158S) and its variants did not alter the expression of proapoptotic proteins such as BAX or PUMA (Supplementary Figure S4).

To examine the effect of PTEN that is frequently deleted or mutated in various kinds of cancers, we ectopically expressed PTEN in PC3 cells and examined cytochrome *c* release. In immunostaining and cell fractionation analyses, cytochrome *c* release was blocked in the cells transfected with a PTEN-expressing vector, in contrast with the cells transfected with an empty vector (Figures 7d and e; Supplementary Figure S5). In fact, production of cleaved PARP, a well-known caspase 3 substrate, was abolished by *PTEN* transfection (Figure 7f). These results imply that BH3_BIM_(I155R/E158S) might be used as apoptosis inducer in PTEN-mutated cancer cells. In contrast with PC3 cells, the viability of HEK293 cells was not affected by the GST-BH3_BIM_-coated AuNPs, which might be ascribed to the normal activity of Akt in HEK293 cells (Figure 7g). Indeed, the AuNP-protein and the CPP-peptide slightly increased cell viability without a statistical significance (Supplementary Figure S6), indicating that the designed peptide is not harmful to untransformed cells. Together, these results demonstrate that it is possible to convert the hyperactivity of Akt in cancer cells into a death signal.

## Discussion

Hyperactivation of Akt is one of the most common molecular perturbations, frequently found in many types of cancers, including glioma, non-small cell lung cancer (NSCLC), ovarian cancer and prostate cancer,^28, 38, 39, 40^ indicating that Akt is an attractive target for cancer therapy. Several Akt inhibitors have been developed and clinically trialed.^39^ However, these inhibitors seem to inevitably accompany side effects, because Akt is also important for the survival of normal cells. We described a new concept and experimental support of converting a cell-survival signal into a cell-killing apoptotic signal with an aim of killing cancer cells without affecting normal cells. These two paradoxical pathways are connected by a peptide designed to harbor the Akt recognition sequence on the potently apoptotic BIM BH3 domain. The peptide was phosphorylated by Akt as we intended, and the phosphorylated peptide interfered with the binding between BCL-2 and the PUMA BH3 domain, most likely because of its potent binding affinity for BCL-2, whereas the weakly interacting unphosphorylated peptide failed to do so. The crystal structures demonstrated how phosphorylation of Ser158 in the peptide significantly enhances the binding affinity of the peptide for BCL-X_L_. Remarkably, the peptide killed cancer cells exhibiting uncontrolled Akt activity, while cells bearing the normal Akt activity were unaffected.

The mode of action of the designed peptide is analogous to that of the tumor suppressor, ARF. The ARF protein leads to p53-dependent cell cycle arrest and apoptosis in response to sustained mitogenic signaling from Myc and Ras.^41^ The *arf* gene is deleted or silenced in numerous cancers, highlighting the important role of ARF as a tumor suppressor.^42^ Like ARF, the designed peptide might block tumor progression. We note that the RxRxx*S/T* motif can also be phosphorylated by RSK and S6K, which promote cell proliferation and growth.^43^ In certain cancer cells, the designed peptide is likely to be phosphorylated by all of the three kinases, which trigger the core Akt-RSK-S6K signaling pathways that are activated at the downstream of protooncogenic receptor protein kinases.^43^

The techniques for the intracellular delivery of proteins and peptides are under active development. Combined with effective techniques, the peptide or protein versions of the designed BIM BH3 variant might be developed into an anticancer drug with greatly reduced side effects. Other deregulated kinases leading to uncontrolled cell growth may also be targeted by embedding different recognition sequences in an apoptogenic BH3 sequence. In conclusion, our results represent a conceptual advance in designing peptide or protein-based cancer therapeutics that are potentiated in cancer cells, but remain dormant in normal cells.

## Materials and Methods

### Plasmids

The procedures for the expression of mouse BCL-X_L_ was described previously.^15^ In brief, a DNA fragment coding for mouse BCL-X_L_ (residues 1–44 and 85–196) was cloned into pProEX-HTa (Invitrogen, Carlsbad, CA, USA). To express the BH3_BIM_ peptide and its variants as a GST-fusion protein in *Escherichia coli*, chemically synthesized DNA fragments encoding human BIM BH3 (residues 145-165 with appropriate mutations; BIONEER, Daejeon, South Korea) were inserted into the pGEX-TEV vector (a modified vector derived from pGEX-4T1) using the *Eco*RI and *Hind*III sites. I153R and E156S mutations were introduced into the vector by site-directed mutagenesis methods based on the overlapping PCR method. DNA fragments encoding full-length human *PUMA* was amplified from a human cDNA library (Life Technologies, Carlsbad, CA, USA) by a standard PCR protocol, and inserted into the pGEX-TEV vector using the *Eco*RI and *Hind*III sites. Akt and PTEN expression vectors were purchased from Addgene (Cambridge, MA, USA; PTEN, Akt-WT and Akt-Myr deposited by Dr William Sellers; Akt-KD (K179M) deposited by Dr Mien-Chie Hung)

### Expression and purification of recombinant proteins

Mouse BCL-X_L_ was purified as reported.^15^ In brief, the protein was expressed in the *E. coli* BL21(DE3) strain (Novagen, Darmstadt, Germany) at 18 °C overnight and purified by using a Ni-NTA column (Qiagen, Venlo, Limburg, Nederland) and a HiTrap Q anion exchange column (GE Healthcare, Little Chalfont, Buckinghamshire, UK). The N-terminal hexahistidine tag was cleaved with TEV protease after a Ni-NTA column purification step. GST-fused BH3_BIM_ variants and mouse PUMA were expressed in *E. coli* and purified with the GST-binding agarose resin (Qiagen) equilibrated with 20-mM Tris buffer (pH 8.0) containing 150-mM NaCl and 2-mM *β*-mercaptoethanol. HA-tagged mouse BCL-2 was expressed from the pcDNA5/FRT/TO vector in HEK293 cells.

### Peptides

The synthetic 21-mer peptides, BH3_BIM_(I155R/E158S), BH3_BIM_(R154S/I155R/E158S), p-BH3_BIM_ (I155R/E158S), p-BH3_BIM_(R154S/I155R/E158S) and the CPP-fused BH3BIM(R154S/I155R/E158S) peptide (YGRRARRRARREIWIAQELRRRGDSFNAYYAR; with the underline indicating the CPP residues) were purchased from Peptron (Daejeon, South Korea). All peptides were modified to have an acetylated amino terminus and an amidated carboxyl terminus.

### Crystallization and structural determination

Purified BCL-X_L_ (4 mg/ml) in a buffer consisting of 20-mM Tris-HCl (pH 8.0), 50-mM NaCl and 1-mM dithiothreitol was mixed with p-BH3_BIM_(I155R/E158S) at a molar ratio of 1 : 3. The crystals of the resulting complex were obtained by the hanging-drop vapor diffusion method at 24 °C by mixing and equilibrating 1 *μ*l of the protein solution, 1 *μ*l of a precipitant solution containing 0.1-M sodium acetate (pH 4.8) and 3.0-M sodium chloride and 0.5 *μ*l of 20% v/v 1,1,1,3,3,3-hexafluoro-2-propanol. Before data collection, the crystals were immersed briefly in a cryoprotectant solution, which was the reservoir solution containing additional 15% glycerol. A diffraction data set at 2.1Å was collected on R-Axis IV++ (Rigaku, Tokyo, Japan) at the Korea Research Institute of Bioscience and Biotechnology, Korea. The data set was processed using the program suit HKL2000.^44^ The structure was determined by the molecular replacement method with the CCP4 version of MolRep^45^ using the structure of the BAD BH3 peptide-bound BCL-X_L_^46^ as a search model. Subsequently, model building and refinement were carried out using the COOT^47^ and CNS^48^ programs. The final model did not include residues 32-44 of BCL-X_L_ and the last two C-terminal residues of p-BH3_BIM_(I155R/E158S) whose electron densities were not observed or very weak.

For crystallization of BCL-X_L_ bound to p-BH3_BIM_(R154S/I155R/E158S), the purified BCL-X_L_ sample (18.5 mg/ml) was mixed with p-BH3_BIM_(R154S/I155R/E158S) at a molar ratio of 1 : 2. The crystals of the resulting complex grew in a precipitant solution containing 0.1- M sodium acetate (pH 4.6), 0.01- M calcium chloride and 15% v/v 2-methyl-2,4-pentanediol. Before data collection, the crystals were immersed briefly in a cryoprotectant solution containing additional 20% glycerol. A diffraction data set at 1.65Å was collected at the 5C beamline of the Pohang Accelerator Laboratory (PAL, Pohang, South Korea) using a QUANTUM 270 CCD detector (ADSC). The data set was processed using the program suit HKL2000.^44^ The structure was determined by the molecular replacement method with the CCP4 version of MolRep^45^ using the structure of the p-BH3_BIM_(I155R/E158S) peptide-bound BCL-X_L_ as a search model. Subsequently, model building and refinement were carried out using the programs COOT^47^ and PHENIX.^49^ Figures were generated using PyMOL.^50^ The final model did not include residues 1 and 31-43 of BCL-X_L_, which are disordered in the crystal. Crystallographic data statistics are summarized in Table 1.

### Isothermal titration calorimetry

All measurements were carried out at 25 ^°^C on a microcalorimetry system iTC200 (GE Healthcare). The BCL-2 and BCL-X_L_ samples were dialyzed against the solution containing 20 mM Tris-HCl (pH 7.5) and 350 mM NaCl. Peptides were titrated into the proteins. Dilution enthalpies were measured in separate experiments (titrant into buffer) and subtracted from the enthalpies of the binding between the protein and the titrant. Data were analyzed using the Origin software (OriginLab Corp., Northampton, MA, USA). We did three independent experiments, which gave the similar results.

### *In vitro* kinase assay for Akt

Each reaction mixture (20 *μ*l) contained recombinant protein substrate (9 *μ*M), Akt/PKBa (0.4 *μ*M) purchased from Millipore (Billerica, MA, USA), ATP (100 *μ*M), and [*γ*-^32^P]ATP (0.5 mCi/ml) in a buffer solution containing 250 mM Tris-HCl (pH 8.0), 50 mM MgCl_2_ and 50 mM dithiothreitol. The mixture was incubated for 1 h at 37 ^°^C and loaded onto a 15% SDS-PAGE gel. After electrophoresis (250 V, 37 min), the radioactivity of the gel was detected with FLA-7000 (Fujifilm, Tokyo, Japan).

### Measurement of cell viability

PC3 and HEK293 cells were incubated with the GST-BH3_BIM_(I155R/E158S) protein conjugated to AuNP-anti-GST DNA aptamer composite^37^ after transfection with a PTEN expression vector (pCMV Flag WT-*PTEN*; addgene plasmid #22231). To examine the effect of the CPP-fused BH3_BIM_ peptide on cell viability, PC3 and HCT116 cells were incubated with the peptide for 48 and 72 h. To examine cell viability, these cells were incubated with 0.5 mg/ml of MTT solution (Calbiochem, Darmstadt, Germany) for 4 h at 37 °C. After removing excess solution and washing with PBS, the precipitated materials were dissolved in 200 ml of dimethyl sulfoxide and quantified by measuring the absorbance at 540 nm. Each experiment was performed by at least three different researchers.

### Immunofluorescence staining

Cells were seeded on a cover glass and incubated with the CCP-fused BH3_BIM_(I155R/E158S) peptide. After fixing with methanol for 30 min, cells were incubated with blocking buffer (0.2% (v/v) normal human antibodies in PBS) for 1 h. After extensive washing with PBS, they were incubated with 1% (v/v) anti-cytochrome *c* mouse antibody (BD, cat# 556432) in blocking buffer for 4 h and subsequently with 0.2% (v/v) FITC-conjugated anti-mouse Ab in PBS containing anti-human antibodies for 2 h. The nucleus was stained by DAPI. After washing with PBS, cover glasses were mounted with Vectamount (Vector Laboratories, Burlingame, CA, USA). The immunofluorescence signal was detected by fluorescence microscopy (Zeiss, Oberkochen, Germany).

### Statistical analysis

Statistical significance was obtained by student's *t*-test.

## Figures and Tables

**Table 1:** Data collection and structure refinement statistics

	**BCL-X_L_−p-BIM_BH3_(I155R/E158S)**	**BCL-X_L_−p-BIM_BH3_(R154S/I155R/E158S)**
Space group	*P*3_2_21	*P*3

*Unit cell dimensions*		
* a*, *b*, *c* (Å)	72.9, 72.9, 75.5	81.7, 81.7, 42.6
* *Wavelength (Å)	1.5418	0.97934
* *Resolution (Å)	50–2.09 (2.13–2.09)^a^	50–1.65 (1.68–1.65)
* R*_sym_^b^	10.6 (23.1)	11.7 (46.6)
* I*/*σ*(*I*)	34.5 (8.4)	17.3 (2.1)
* *Completeness (%)	96.4 (77.5)	99.2 (94.2)
* *Redundancy	6.3	6.4

*Refinement*		
* *Resolution (Å)	50.0–2.1	20.0–1.7
* *Number of reflections	13580	34825
* R*_work_^c^/*R*_free_	18.3/21.1	19.2/22.8

*Number of atoms*		
* *Protein	1364	2701
* *Water	143	117
* *Ion	6	6

*R.M.S deviations*		
* *Bond lengths (Å)	0.005	0.007
* *Bond angles (^o^)	1.050	1.777

*Ramachandran plot (%)*		
* *Most favored region	95.3	99.4
* *Additionally allowed region	4.7	0.6

*Average B-values (Å*^*2*^)		
* *Protein	18.4	12.3
* *Peptide	18.8	11.0
* *Water	27.7	19.2

a The numbers in parentheses are statistics from the highest resolution shell.

b *R*_sym_=Σ |*I*_obs_−*I*_avg_| / *I*_obs_, where *I*_obs_ is the observed intensity of individual reflection and *I*_avg_ is average over symmetry equivalents.

c *R*_work_=Σ ||*F*_o_|−|*F*_c_|| / Σ |*F*_o_|, where |*F*_o_| and |*F*_c_| are the observed and calculated structure factor amplitudes, respectively. *R*_free_ was calculated with 5% of the data

## Abbreviations

BH: BCL-2 homology
MOM: mitochondrial outer membrane
ITC: isothermal titration calorimetry
AuNP: gold nanoparticle
NSCLS: non-small cell lung cancer
