# Supplementary material for: Conversion of cell-survival activity of Akt into apoptotic death of cancer cells by two mutations on the BIM BH3 domain
Source: Cell Death Dis. 2015 Jul 2;6(7):e1804–. doi: 10.1038/cddis.2015.118 (PMC4650712; doi:10.1038/cddis.2015.118)

**Supplemental Figure Legends**

**Figure S1. Structure of BCL-X_L_ bound to p-BH3_BIM_(I155R/E158S) highlighting the crystal packing interactions of the Arg154 of the peptide.** The hydrogen-bonding network with the neighboring molecule (BCL-X_L_’) is highlighted in grey.


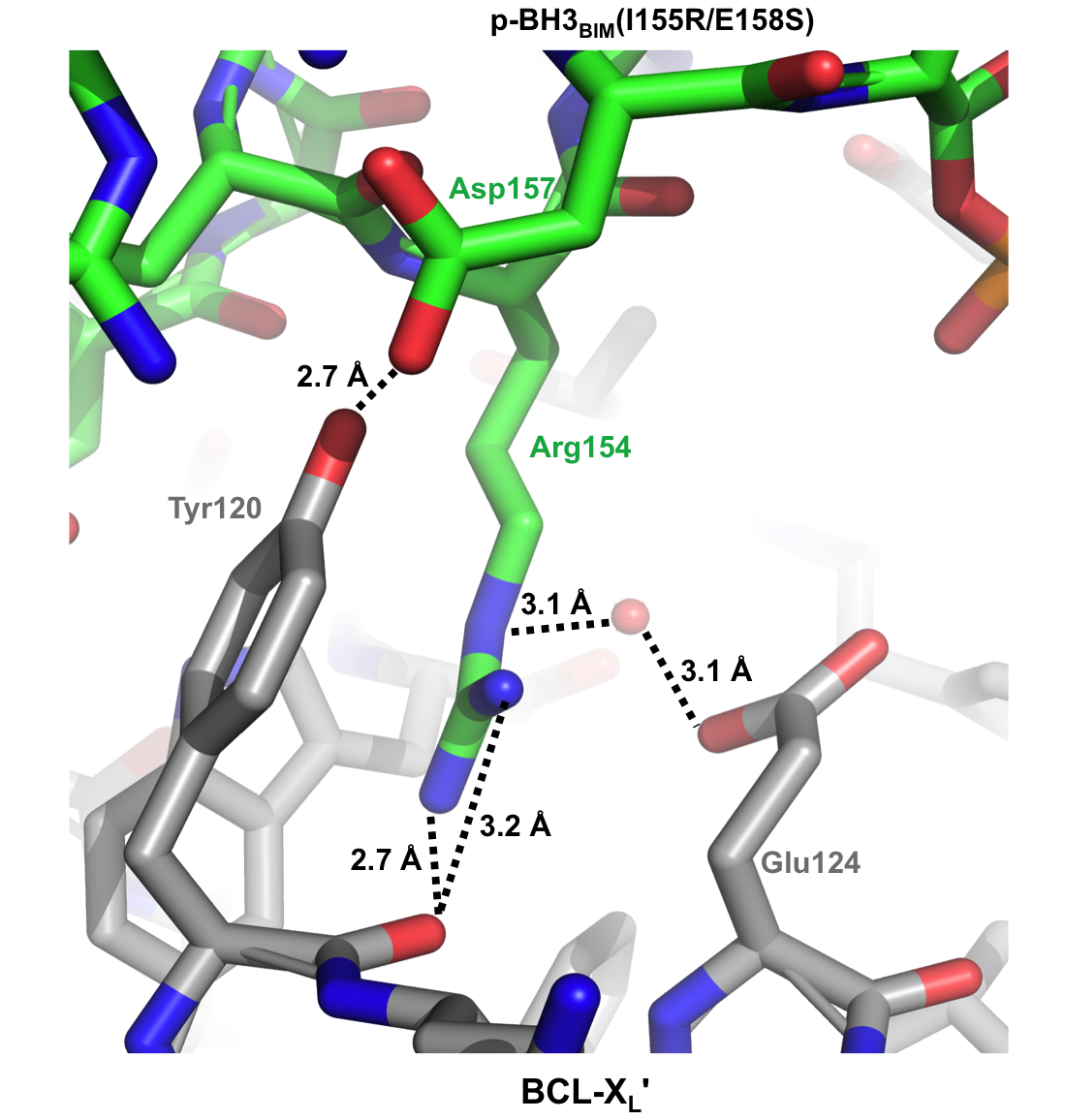


**Figure S2. ITC analysis of the interaction between BCL-X_L_ and p-BH3_BIM_(R154S/I155R/E158S)**

The analysis was carried out by titrating p-BH3_BIM_(R154S/I155R/E158S) (0.2 mM) into BCL-X_L_ (20 μM). The *K*_D_ values were deduced from the curve fittings of the integrated heat per mole of added ligand (insets).


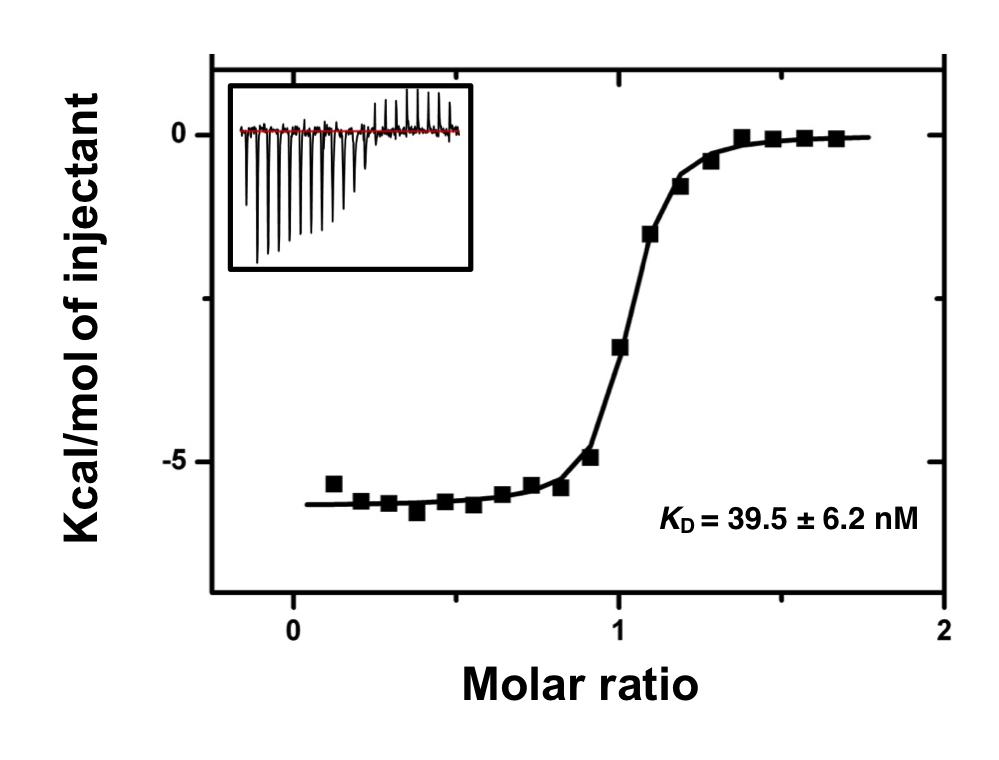


**Figure S3. Structure of BCL-X_L_ bound to p-BH3_BIM_(R154S/I155R/E158S).**

(A) Two molecules of the complex in the asymmetric unit of the crystal. BCL-X_L_ is in red and the peptide is in green.

(B) Structural superposition of the two molecules of the complex in the asymmetric unit.


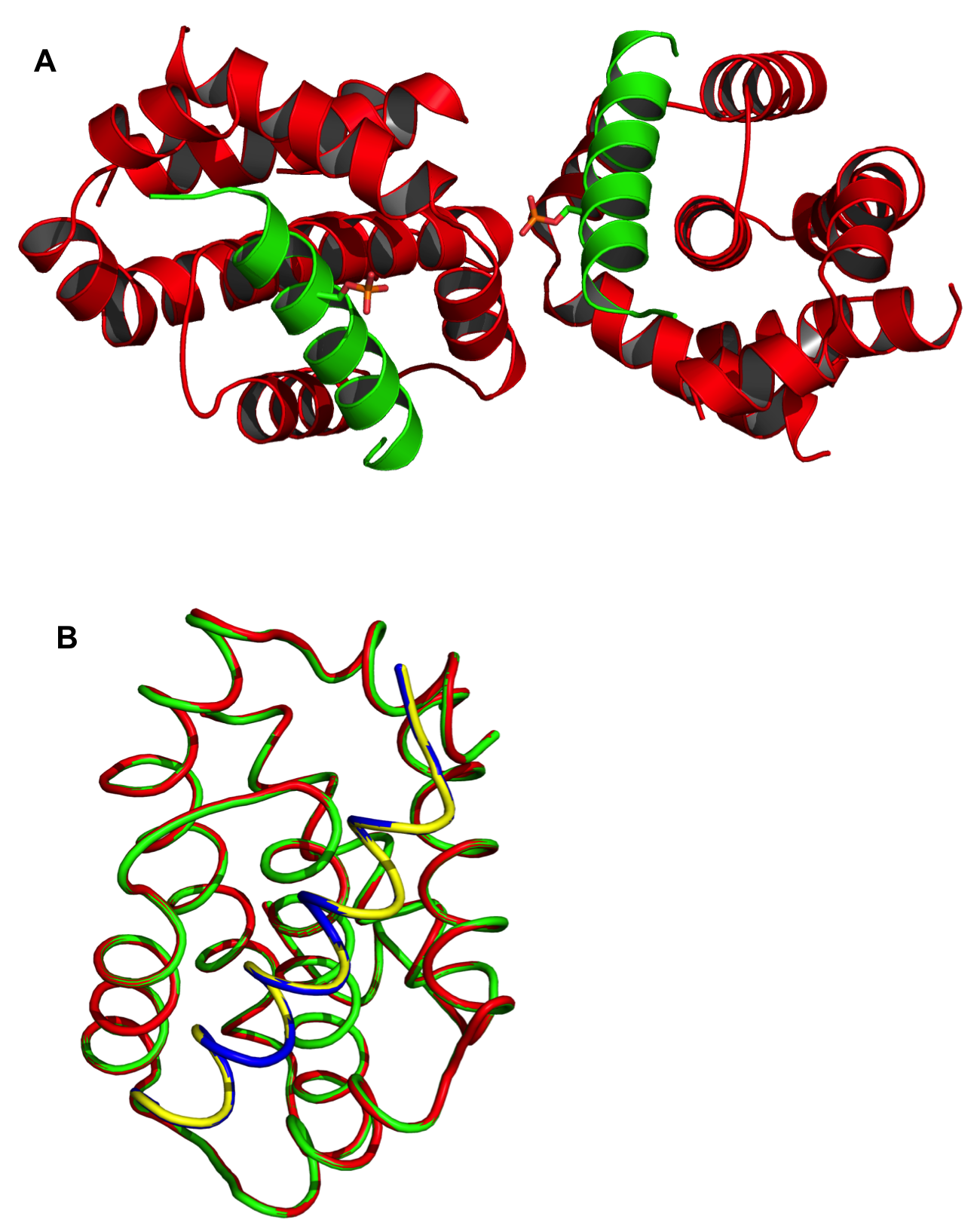


**Figure S4. AuNP and AuNP-GST-BIM did not alter the BAX or PUMA expression in PC3, in regardless of apoptotic activity**. PC3 cells were incubated with indicated materials for 24 hr.


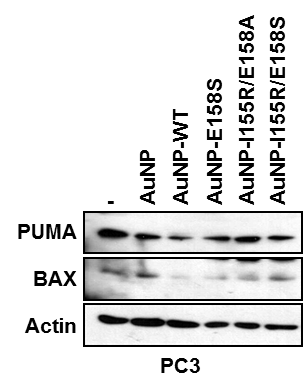


**Figure S5. Cytochrome *c* release in PTEN transfected cells.** Although large portion of PC3 were expressed PTEN (red) by transfection, several untransfected cells were found, where cytochrome *c* (green) was diffused into nuclei (arrows). DAPI stained nuclear DNA (blue).


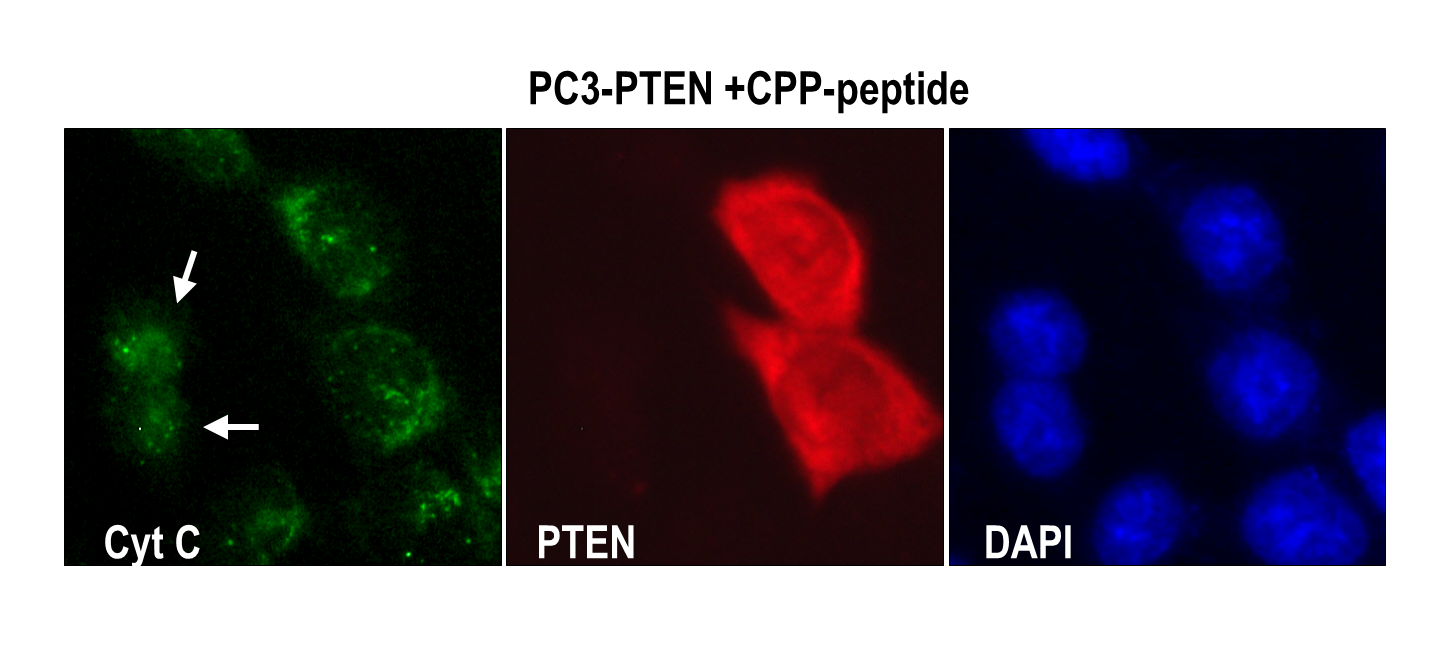


**Figure S6. Slight increase of cell viability by CPP-peptide in 293.** Consistently with Fig. 7G, treatment of BH3_BIM_ slightly increases cell viability that was measured by MTT.


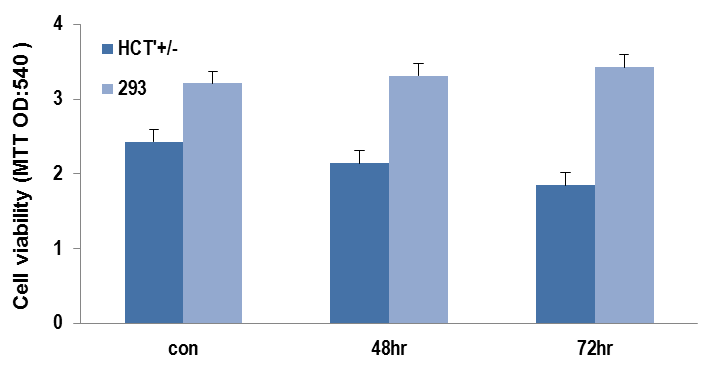

Supplement: Supplementary Figures [file cddis2015118x1.docx]
